# The pattern of methacholine responsiveness in mice is dependent on antigen challenge dose

**DOI:** 10.1186/1465-9921-5-15

**Published:** 2004-09-23

**Authors:** Graeme R Zosky, Christophe von Garnier, Philip A Stumbles, Patrick G Holt, Peter D Sly, Debra J Turner

**Affiliations:** 1 Telethon Institute for Child Health Research, West Perth, 6872, Australia; 2 Centre for Child Health Research, University of Western Australia, Crawley, 6009, Australia

## Abstract

**Background:**

Considerable variation exists in the protocols used to induce hyperresponsiveness in murine models of allergic sensitisation. We examined the effect of varying the number of antigen exposures at challenge on the development of methacholine responsiveness in systemically sensitised mice.

**Methods:**

BALB/c mice were sensitised with ovalbumin (OVA), challenged with 1, 3 or 6 OVA aerosols. Lung function was measured using low frequency forced oscillations and partitioned into components representing the airways (R_aw_) and lung parenchyma (tissue damping (G) and tissue elastance (H)). Responsiveness to inhaled methacholine (MCh), inflammatory cell profile and circulating IgE were assessed 24 and 48 hours after challenge. The threshold dose of MCh required to elicit a detectable response (sensitivity) and response to 30 mg.mL^-1 ^(maximal response) were determined for each compartment.

**Results:**

*Sensitivity*; All three OVA protocols resulted in an increased sensitivity to MCh in R_aw _but not in G or H. These responses where present at 24 and 48 hrs, except 1 OVA aerosol in which changes had resolved by 48 hrs. *Maximal response*; 1 OVA aerosol increased maximal responses in R_aw_, G and H at 24 hrs, which was gone by 48 hrs. Three OVA aerosols increased responses in H at 48 hrs only. Six OVA challenges caused increases in R_aw_, G and H at both 24 and 48 hrs. Eosinophils increased with increasing antigen challenges. IgE was elevated by OVA sensitisation but not boosted by OVA aerosol challenge.

**Conclusions:**

The pattern of eosinophilia, IgE and MCh responsiveness in mice was determined by antigen dose at challenge. In this study, increased sensitivity to MCh was confined to the airways whereas increases in maximal responses occurred in both the airway and parenchymal compartments. The presence of eosinophilia and IgE did not always coincide with increased responsiveness to inhaled MCh. These findings require further systematic study to determine whether different mechanisms underlie airway and parenchymal hyperresponsiveness post antigen challenge.

## Background

Persistent asthma is an allergic disease characterised by airway inflammation ([1-5]) and hyperresponsiveness to external stimuli ([1]). Mouse models of allergic airway sensitisation are often used to elucidate the pathobiology of this disease ([6-8]).

To date, a number of techniques have been used to measure changes in lung function in response to bronchoconstricting agents in murine models of allergic bronchopulmonary inflammation (see [6,8,9] for reviews). One method that has gained recent popularity is unrestrained barometric plethysmography, which uses a 'pseudo-flow' measurement to derive a dimensionless parameter known as enhanced pause (Penh). There are now several publications in the literature which claim to have documented airway hyperresponsiveness in allergen-driven murine models based on methacholine induced changes in Penh. However, it has also been well documented that Penh does not correlate with changes in the physiology of the lung ([10-14]), especially in chronic disease states ([15]). In contrast, the low frequency forced oscillation technique (LFOT) is able to provide sensitive measurements of respiratory system input impedance (Zrs) in the mouse, that are partitioned into components representing airway and parenchymal compartments by fitting the constant-phase model ([16-18]). Using LFOT, Tomioka *et al*. ([17]) found that systemic sensitisation followed by three antigen challenges, one of the most common allergen models utilised in studies using Penh, resulted in hyperresponsiveness that was confined primarily to the tissue compartment of the lung. This has important implications for the interpretation of results obtained with Penh that have demonstrated mechanisms underlying allergic inflammation in mice given that a significant portion of the respiratory system hyperreactivity to MCh in human asthmatics is a result of the response of the conducting airways ([19]).

One of the most common methods for inducing allergic bronchopulmonary inflammation in mice involves systemic sensitisation with a specific antigen and Th-2 skewing adjuvant, usually ovalbumin (OVA) adsorbed onto aluminium hydroxide (Alum), followed by airway challenge with the same antigen ([20-22]). However, considerable variations exist between studies in terms of the dose of antigen used during airway challenge. To date, a number of studies have found that airway hyperresponsiveness is increased by increasing the dose of antigen at challenge ([23-25]). However, these studies, which used different doses of antigen at challenge as part of a broader intervention protocol, have used Penh ([23,24]) or a measure of total lung resistance ([25]) to examine the resulting changes in lung physiology. As yet, no studies have systematically examined the effect of the dose of antigen at challenge on the subsequent development of hyperresponsiveness using a technique that is able to partition the reactivity of the lungs into airways and tissue compartments.

Hyperresponsiveness of the respiratory system to bronchoconstricting agents, and other outcome parameters such as those that reflect inflammation and allergic sensitisation, are often measured at different times post challenge. In an examination of the kinetics of hyperresponsiveness in an OVA model of allergic sensitisation in mice using a single dose of antigen at challenge, Tomkinson *et al*. ([26]) found that responsiveness to methacholine (MCh) is maximal 24 hours post challenge, has begun to resolve by 48 hours, and has returned to baseline levels beyond that time. The kinetics of responsiveness to MCh in other studies, however, are often overlooked and it is yet be determined if altering the dose of antigen at challenge has an influence on the timing of peak responsiveness to bronchoconstricting agents.

The aim of this study was to systemically investigate the effect of antigen dose at challenge on the pattern of hyperresponsiveness to inhaled MCh in a murine model of allergic bronchopulmonary inflammation.

## Methods

### Animals

8 week old specific pathogen free female BALB/c mice were purchased from the Animal Resources Centre, Murdoch, Western Australia. Mice were housed in a controlled environment with a 12 hr light:dark cycle and provided with an OVA free diet and acidified water *ad libitum*. All experiments were approved by the Institutional Animal Ethics and Experimentation Committee.

### Sensitisation protocols

**Figure 1:**
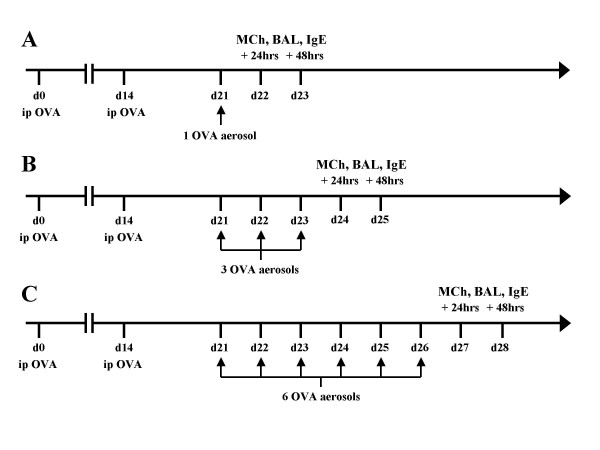
**Timeline for sensitisation and data collection. **Timeline for the protocols used to induce allergic bronchopulmonary inflammation and timing for bronchoalveolar lavage (BAL), serum IgE measurement and assessment of hyperresponsiveness to inhaled methacholine (MCh). Mice were systemically sensitised with two intraperitoneal injections of OVA/Alum on day 0 and 14, challenged with either 1 (A), 3 (B) or 6 (C) OVA aerosols (1%) for 30 minutes starting at day 21.

Mice were sensitised by intraperitoneal (i.p.) injection with 20 μg of OVA (Sigma, St Louis, USA) suspended in 200 μL of Alum (Alu-gel-S, Serva, Heidelberg, Germany) on days 0 and 14. Mice were then challenged with either 1, 3 or 6 OVA (1% w/v in PBS) aerosols delivered with an ultrasonic nebuliser (UltraNeb^®^, DeVilbiss, Somerset, Pennsylvania) for 30 minutes on consecutive days starting at day 21 (Fig 1). Two additional groups of mice served as controls; a naïve group and a group sensitised with i.p. OVA and challenged with a single PBS aerosol using the protocol described above.

### Respiratory mechanics

Changes in Zrs were measured using a modification of the low frequency forced-oscillation technique (LFOT) as described previously ([27]). Briefly, mice were anaesthetised with an i.p. injection of a solution containing xylazine (2 mg.mL^-1^, Troy Laboratories, NSW, Australia) and ketamine (40 mg.mL^-1^, Troy Laboratories, NSW, Australia) at a dose of 0.01 mL.g^-1^. Mice were tracheostomised with a 10 mm section of polyethylene tubing (1.27 mm OD: 0.86 mm ID) and ventilated (*flexiVent*, Scireq, Montreal, Canada) at 450 b.min^-1 ^with a tidal volume of 8 mL.kg^-1 ^and a positive end expiratory pressure (PEEP) of 2 cmH_2_O. The lung volume history of the mice was standardised prior to measurement of lung mechanics using two deep inflations and three P-V curves. The respiratory system input impedance (Zrs) was measured during periods of apnea using a 16 s signal containing 19 mutually prime sinusoidal frequencies ranging from 0.25 to 19.625 Hz. The constant phase model ([16]) was then fit to the real and imaginary parts of the Zrs spectrum allowing the calculation of airway resistance (R_aw_), tissue damping (G), tissue elastance (H) and hysteresivity (η) ([28]).

### Methacholine responsiveness

Changes in respiratory mechanics following inhaled MCh were measured either 24 or 48 hrs after the last OVA aerosol. Following measurement of baseline Zrs, mice were exposed to a 90 s saline aerosol delivered with an ultrasonic nebuliser (UltraNeb^®^, Devilbiss, Somerset, Pennsylvania). Zrs was then measured every minute for the next 5 minutes. This aerosol procedure was repeated with 1/2 log_10 _incremental doses of MCh from 0.1 to 30 mg.mL^-1 ^with Zrs measured every minute for at least 5 minutes after the aerosol until the parameters calculated from the constant phase model had peaked.

### Inflammatory cell counts

Separate groups of mice, sensitised using the same protocol described above, were anaesthetised and tracheostomised 24 or 48 hrs after their last aerosol. BAL fluid was collected by slowly infusing and withdrawing a 1 mL aliquot of PBS containing BSA (bovine serum albumin, 20 mg.mL^-1^, CSL, Victoria, Australia) and lidocaine (35 mg.mL^-1^, Sigma, St Louis, USA) from the lungs three times. The BAL was then centrifuged at 2000 rpm for 4 mins. The supernatant was removed and the pellet resuspended in PBS. The cells were stained with trypan blue to determine viability and the total cell count (TCC) obtained by counting the cells with a haemocytometer. Differential counts were obtained from the cytospin sample, stained with Leishman's stain and examined using light microscopy. Three hundred cells were counted from each sample to determine the relative proportions of each cell type.

### Serum IgE

In a separate group of mice, serum samples were periodically collected for analysis of total IgE. An additional control group was included in the analysis of serum IgE consisting of mice sensitised with PBS/Alum. Sera were diluted 1:7.5 in Delfia Assay buffer (Wallac Oy, Turku, Finland). The diluted sera were analysed for the presence of total IgE by time-resolved fluorescence (TRF) assays. Briefly, 96-well plates (Nunc Maxisorp, Denmark) were coated overnight at 4° C with anti-mouse IgE (R35-72; BD PharMingen, San Diego, USA). Plates were blocked with 200 μl of 0.5% BSA in TRIS-HCl pH 7.4 for 1 hour at room temperature on a plate shaker. For all subsequent steps a volume of 50 μl per well was used and incubations were performed for 1 hour at room temperature unless otherwise indicated. Between steps, plates were washed five times with wash buffer (TRIS-HCl pH 7.8 Tween20). Mouse anti-TNP IgE (BD PharMingen, San Diego, USA) was used as an interassay standard. Biotinylated anti-mouse IgE (R35-118; BD PharMingen, San Diego, USA) was added to the wells at 2 μg.mL^-1^. Straptavidin-conjugated Europium (Wallac Oy, Turku, Finland) was incubated at 1:500 for 30 minutes and plates washed eight times thereafter. Delfia enhancement solution (Wallac Oy, Turku, Finland) was added and the plates were agitated on a shaker for 10 minutes prior to reading the fluorescence on a Wallac Victor 2 counter (Wallac Oy, Turku, Finland). The detection limit of this assay is approximately 100 ng.mL^-1^.

### Statistical analysis

**Figure 2:**
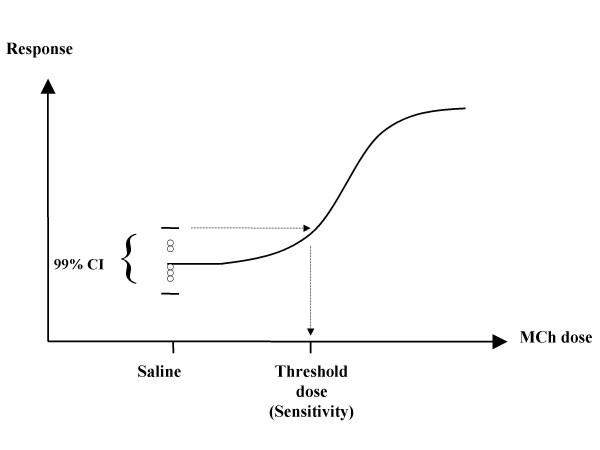
**Technique for sensitivity calculation. **Schematic representation of the technique used for calculation of the threshold dose of MCh (sensitivity) required to induce a detectable increase in R_aw_, G and H.

Log_10 _transformed inflammatory cell and immunoglobulin data were compared using ANOVA and Tukey's post-hoc test. Responses in R_aw _and G to inhaled MCh at the maximum dose used (30 mg.mL^-1^) were expressed as a percentage of the response to the saline aerosol and compared using non-parametric ANOVA on ranks and Dunn's post-hoc test. Responses in H were expressed as a percentage of the response to saline, log_10 _transformed and compared using ANOVA and Tukey's post-hoc test. The threshold dose of MCh where there was a detectable change in R_aw_, G or H (termed sensitivity hereafter) was interpolated from the raw dose response curve as the upper limit of the 99% CI of the 5 measurements taken following the saline aerosol (Fig. 2). The sensitivity data were compared using ANOVA and Tukey's post-hoc test. All data were analysed using SigmaStat 2.03 and p values < 0.05 were deemed to be significant.

## Results

### Methacholine responsiveness

**Figure 3:**
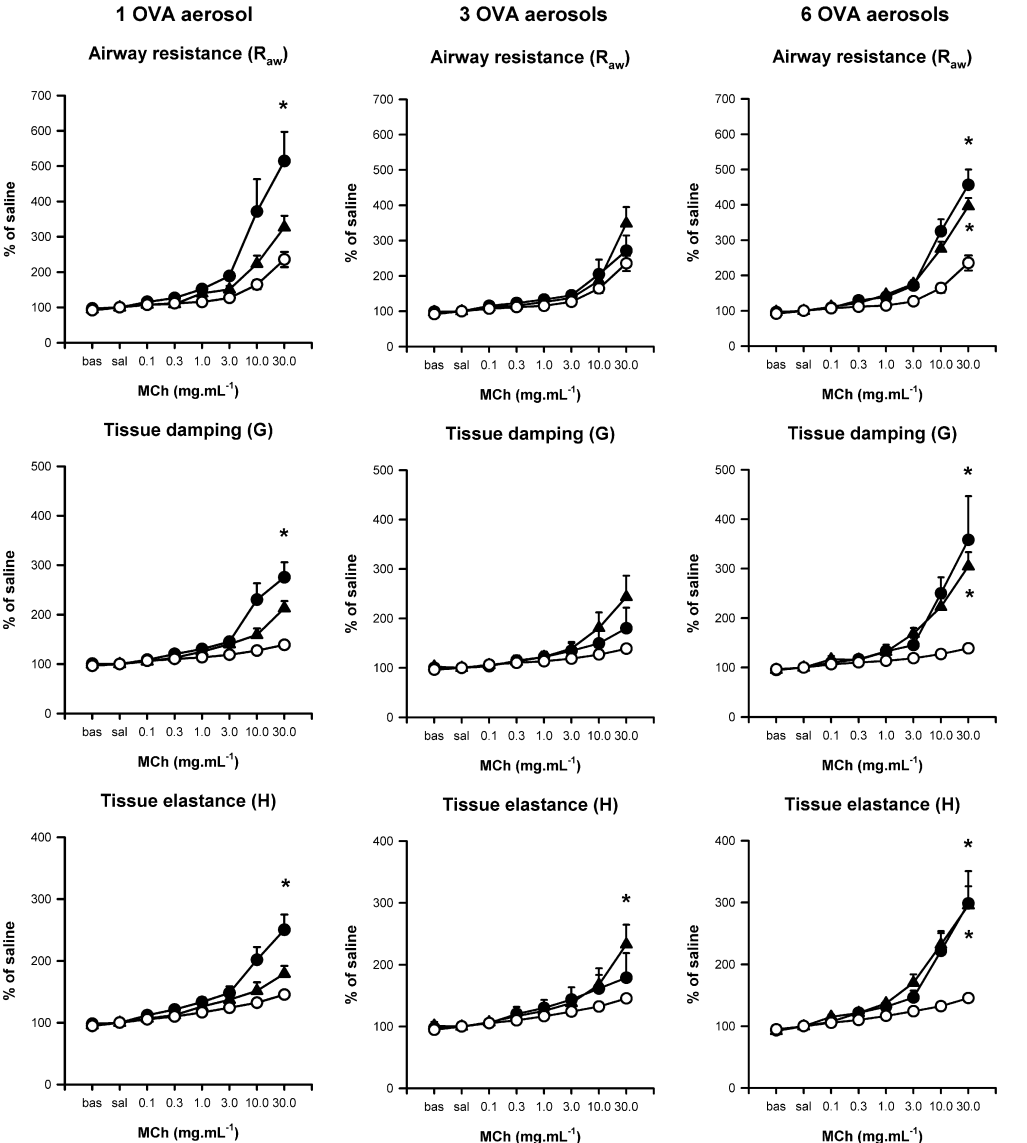
**Dose response curves to inhaled methacholine. **Dose response curves (expressed as a % of the response to saline aerosol) for mice systemically sensitised with OVA/Alum and challenged via the airways with 1 (left), 3 (centre) or 6 (right) OVA aerosols. Mice were challenged 24 (●) or 48 (▲) hours after the last OVA aerosol. Dose response curves from naïve mice (○) are also shown. All data are expressed as mean ± SEM (n = 7–8). * indicates significance (p < 0.05 vs naïve mice; ANOVA on Ranks, Dunn's post-hoc for R_aw _and G; ANOVA, Tukey's post-hoc for H).

The degree and time of observed maximum MCh induced responses in R_aw_, G and H varied substantially between treatments (Fig. 3). A summary of statistical comparisons of sensitivity to MCh and percentage response to the maximum dose (30 mg.mL^-1^) between treatment groups and naïve mice is presented in Table 1. Sensitisation followed by challenge with a single PBS aerosol did not cause an increase in sensitivity or maximum responsiveness to MCh compared to naïve mice.

**Table 1:** Summary of sensitivity and maximum responses to methacholine in airway and parenchymal lung compartments. Summary of the threshold dose (sensitivity) required to elicit a detectable increase in airway resistance (R_aw_), tissue damping (G) and tissue elastance (H) for naïve mice, mice systemically sensitised with OVA/Alum and challenged with PBS and mice systemically sensitised and challenged with OVA. Also shown is the percentage change in R_aw_, G and H in response to the maximum does of methacholine used (30 mg.mL^-1^). Data are presented as the mean (SEM).

**Challenge**	**Assessed after last aerosol (hr)**	**Sensitivity - Threshold dose of MCh (mg.mL^-1^)**						**Response at 30 mg.mL^-1 ^MCh**					
		**Raw**		**G**		**H**		**Raw^§^**		**G^§^**		**H^§^**	
			p*		p*		p*		p*		p*		p*
Naïve	-	0.54(0.14)	-	0.51(0.25)	-	0.10(0.02)	-	235.5(21.5)	-	138.8(6.1)	-	145.4(5.1)	-
1 PBS aerosol	24 and 48 pooled	0.55(0.30)	ns	0.25(0.10)	ns	0.06(0.01)	ns	242.9(19.1)	ns	141.5(4.7)	ns	143.4(2.7)	ns
1 OVA aerosol	24	0.09(0.03)	**0.012**	0.15(0.04)	ns	0.05(0.01)	ns	514.0(82.7)	**<0.05**	275.5(30.6)	**<0.001**	250.2(24.5)	**<0.001**
	48	0.46(0.19)	ns	0.18(0.05)	ns	0.17(0.07)	ns	326.4(32.6)	ns	213.0(14.6)	ns	179.3(12.6)	ns
3 OVA aerosols	24	0.12(0.03)	**0.012**	0.35(0.20)	ns	0.06(0.01)	ns	271.2(43.1)	ns	180.3(41.7)	ns	178.9(39.9)	ns
	48	0.20(0.07)	**0.034**	0.16(0.04)	ns	0.08(0.01)	ns	348.8(46.0)	ns	243.6(42.7)	ns	233.0(31.6)	**0.045**
6 OVA aerosols	24	0.17(0.05)	**0.019**	0.12(0.05)	ns	0.06(0.01)	ns	456.7(43.4)	**<0.05**	358.2(88.4)	**<0.05**	298.5(52.2)	**0.02**
	48	0.16(0.05)	**0.034**	0.15(0.06)	ns	0.06(0.01)	ns	396.0(23.3)	**<0.05**	304.8(28.5)	**<0.05**	295.6(30.6)	**0.018**

§ expressed as a % of saline response

* vs naïve values

#### One OVA aerosol

A single OVA aerosol was sufficient to induce a significant increase in MCh responsiveness in the airways, seen as both a lower threshold dose of MCh required to induce a response (increased sensitivity) and increased response at the 24 hour time point (Table 1). In the parenchymal compartment, no increase in sensitivity was seen but a significant increase in maximal response was seen for both G and H. This heightened sensitivity and response had diminished, back to the level seen in naive mice, 48 hours after the OVA aerosol.

#### Three OVA aerosols

Three OVA aerosols resulted in significantly increased airway (but not parenchymal) sensitivity to MCh at both the 24 and 48 hour time points (Table 1). However, there was no increase in maximum response at 24 hours in R_aw_, G or H and only an increased response in H after 48 hours but not R_aw _and G.

#### Six OVA aerosols

Six OVA aerosols resulted in both significantly increased airway sensitivity and maximal responses to MCh at 24 and 48 hours post-challenge. Increased maximal responses, but not increased sensitivity, were also seen in the parenchymal compartment at both the 24 and 48 hour time points.

### Inflammatory cell counts

**Figure 4:**
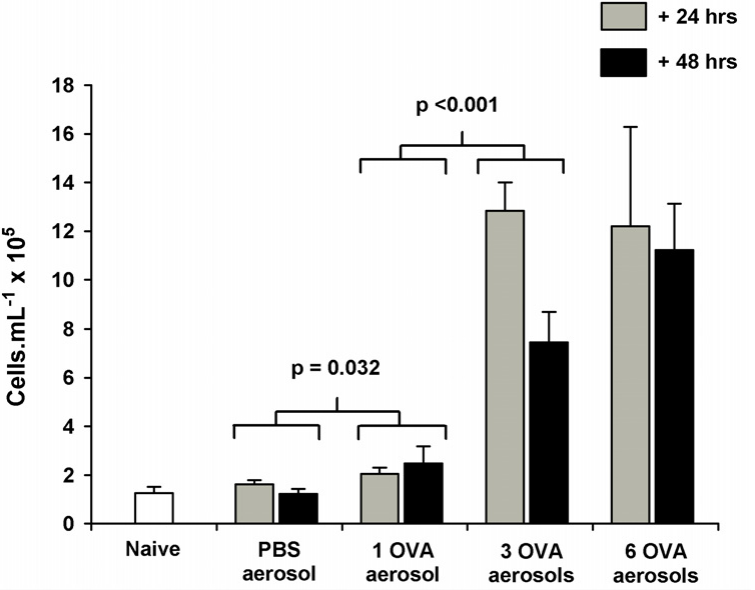
**Total cell counts from bronchoalveolar lavage. **Total cell counts (TCC) from the bronchoalveolar lavage (BAL) of naïve BALB/c mice, mice systemically sensitised and challenge with OVA aerosols and mice systemically sensitised with OVA and challenged with PBS. Samples were collected 24 (grey) and 48 (black) hours after the last aerosol. Data are expressed as mean ± SEM (n = 5–6). Exposure to a PBS aerosol following antigen sensitisation did not cause an increase in TCC (p = 0.552). In contrast, a single OVA aerosol was sufficient to cause a significant increase in TCC (p = 0.032). Exposure to 3 OVA aerosols caused a further increase in TCC (p < 0.001) but 6 OVA aerosols did not cause an increase in TCC beyond those observed in mice exposed to 3 OVA aerosols (p = 0.805).

Challenge with a single PBS aerosol following systemic sensitisation with OVA did not cause a significant increase in TCC in the BAL (p = 0.552) compared to naïve mice (Fig. 4). There was, however, a significant increase in TCC in mice challenged with a single OVA aerosol (p = 0.032) and a further increase in TCC following 3 OVA challenges (p < 0.001). Exposure to 6 OVA aerosols did not cause any further increase in TCC above levels observed in mice exposed to 3 OVA aerosols (p = 0.805) but remained significantly higher than mice challenged with 1 OVA aerosol (p < 0.001). Time of sampling after the last aerosol with any of the protocols did not have a significant impact on TCC (p = 0.357).

**Figure 5:**
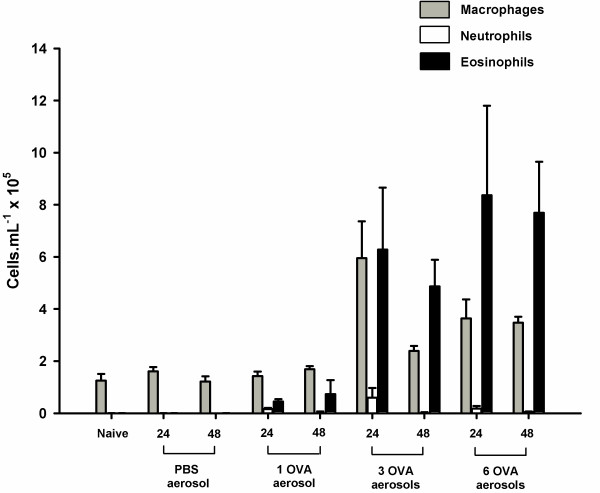
**Differential cell counts from bronchoalveolar lavage. **Differential cell counts from the bronchoalveolar lavage (BAL) of naïve BALB/c mice, mice systemically sensitised and challenge with 1,3 or 6 OVA aerosols and mice systemically sensitized with OVA and challenged with a single PBS aerosol. BALs were collected 24 and 48 hours after the last aerosol. Data are expressed as mean ± SEM (n = 5–6). There was a significant increase in the number of eosinophils (p = 0.032) in the BAL following a single OVA aerosol. Exposure to 3 or more OVA aerosols caused a further increase in the number eosinophils (p < 0.001), compared to 1 OVA aerosol, and an increase in the number of macrophages (p < 0.001) compared to naïve mice. There were neutrophils present in the BALs of some mice but only in those groups sensitised and challenged with OVA and only in BALs sampled 24 hours after the last aerosol.

The number of aeroallergen challenges also had a significant impact on the number of eosinophils (p < 0.001) and macrophages (p < 0.001) in the BAL. There were significant increases in the number of eosinophils in sensitised mice challenged with 1 (p = 0.032), 3 (p < 0.001) and 6 (p < 0.001) OVA aerosols (Fig. 5) compared to naïve mice. The numbers of eosinophils in the BAL of mice exposed to 3 and 6 aerosols were significantly higher than those exposed to a single OVA aerosol (p < 0.001 and p < 0.001 respectively) but were not significantly different from each other (p = 0.805). The number of macrophages in the BAL were also higher in mice exposed to 3 (p < 0.001) and 6 (p < 0.001) OVA aerosols compared to naïve mice. As with TCC, time of sampling after the last aerosol did not have a significant impact on the number of eosinophils (p = 0.357) or macrophages (p = 0.079) in the BAL. Low levels of neutrophils were observed in BALs from OVA challenged mice sampled at 24 hours but not in mice sampled 48 hours after the last OVA aerosol (Fig. 5). Lymphocyte numbers were not significantly elevated in the BALs from any of the treatment groups (*data not shown*).

### Serum IgE

**Figure 6:**
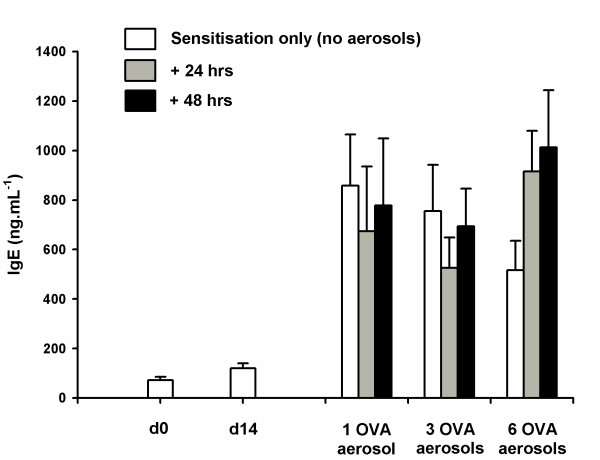
**Total serum IgE obtained from time resolved fluorescence. **Total IgE obtained from time resolved fluorescence assay of serum collected from systemically sensitised (i.p. OVA/Alum on day 0 and day 14) but not challenged with aerosolised antigen (white bars). The vertical bars represent total serum IgE from mice sensitised and challenged with either 1, 3 or 6 OVA aerosols. Serum samples from these mice were collected 24 (grey bars) and 48 (black bars) hours after the last aerosol. Data are expressed as mean ± SEM (n = 10). Two intraperitoneal injections of OVA/Alum were sufficient to induce increased levels total IgE by day 21 (p < 0.001) compared to naïve mice. Exposure to OVA aerosol challenges did not cause a further increase in total IgE (p = 0.842).

Total serum IgE was significantly increased at day 21 (p < 0.001), 7 days after the second injection of OVA/Alum, compared to naïve mice (Fig. 6). In contrast, serum IgE levels at day 14, after a single injection, were not significantly elevated (p = 0.438) compared to naïve mice. The total serum IgE response to systemic sensitisation, in the absence of subsequent antigen aerosol challenge, peaked at day 22 and partially declined by day 27. However, this decrease was not statistically significant (p = 0.511). There was no further increase in the total serum IgE in mice that were sensitised and subsequently challenged with OVA aerosols compared to those that were only systemically sensitised (p = 0.842). Total serum IgE levels were not significantly greater in mice sensitised with PBS/Alum and challenged with OVA (*data not shown*).

## Discussion

Varying the number of aeroallergen challenges in a systemically sensitised murine model of allergic bronchopulmonary inflammation altered the degree and timing of hyperresponsiveness to inhaled MCh. A single OVA challenge increased airway sensitivity to inhaled MCh 24 hours after the challenge, while sensitivity remained elevated for 48 hours after three and six challenges. OVA challenge did not increase parenchymal sensitivity at any level. In contrast to sensitivity measurements, the maximum response to 30 mg.mL^-1 ^MCh showed a variable pattern. A transient response was observed in both airway and parenchymal compartments after a single OVA aerosol. After 3 OVA aerosols significant increases were seen in the tissue compartment at 48 hours, while after 6 OVA aerosols an elevated response was seen in the airway and parenchymal compartments that persisted beyond 48 hours. There was a significant influx of inflammatory cells in the BAL in response to OVA aerosols, however, the presence of this inflammation did not always result in hyperessponsiveness to inhaled MCh.

Murine models using 2 systemic allergen sensitisations followed by 3 aeroallergen challenges are prevalent in the literature ([20,29-31]) and have been reported to demonstrate airway hyperresponsiveness to MCh. However, these studies have used enhanced pause (Penh), which is derived from unrestrained barometric plethysmography, to measure changes in lung physiology. As Penh cannot differentiate between constriction in the airways and changes in the tissue compartment of the lungs, it is impossible to tell where the responses to MCh are localised, if indeed they are true physiological responses ([10-14]). In contrast, our study, using 2 systemic sensitisations and 1,3 or 6 challenges, has demonstrated clear airway, tissue or mixed compartment responses to methacholine which is dependent on the number of aerosol challenges delivered. In our hands, the more common model of 2 systemic sensitisations followed by 3 OVA challenges resulted in increased responsiveness to the maximum dose of MCh that was confined to the tissue compartment of the lung. This finding is consistent with a previous study by Tomioka *et al*. ([17]), which also used a forced oscillation technique to measure changes in lung mechanics in OVA sensitised and challenged mice. The fact that the response was confined to the tissues is of interest as the aim of these models is to mimic the human asthmatic condition, in which a significant portion of reactivity of the lungs is localised in the conducting airways ([19]). This work emphasises the importance of measuring bronchoconstriction with physiological techniques capable of compartmentalising responses within the lungs. By varying the antigen dose at challenge we have revealed a system with the potential to allow investigation of transient or prolonged responsiveness to MCh that is localised in the airways, tissues, or both. Further investigation is needed in order to understand the mechanisms that are influencing the site of responsiveness.

Typically, most human studies measure MCh responsiveness in terms of sensitivity as they report the concentration of MCh required to produce a 20% fall in FEV_1_. We have shown that it is possible to determine sensitivity to inhaled MCh in mice and that only the airway compartment shows heightened sensitivity following allergic sensitisation and challenge. While increased maximal responses can be seen in both airway and parenchymal compartments, depending on which model is used, no increase in parenchymal sensitivity is seen with any of the models we used. As such, these findings reinforce the value of using lung function techniques that are capable of assessing airway and parenchymal mechanics separately.

Total serum IgE was significantly elevated following systemic sensitisation but was not increased by aerosol challenge. There was, however, a tendency for total serum IgE to decline by day 27 in mice that were systemically sensitised but not challenged with OVA aerosols, compared to mice additionally exposed to 6 OVA aerosols. It is possible that if the study had been extended to include further exposure to antigen over subsequent days, a difference would have been detected between mice that were only sensitised and mice that were sensitised and challenged. Given that antigen specific IgE and other immunoglobulin subtypes were not measured in this study, further work is required to characterise the effect of dose of antigen at challenge on the development of antibody responses to OVA in mice.

The protocol used in the present study induced significant eosinophilia after a single airway challenge. The degree of eosinophilia increased with increasing number of airway challenges. This finding is consistent with several previous studies using similar protocols to induce allergic inflammation in the lungs of mice ([20,29-31]). While the level of activation of the eosinophils was not measured in the present study, the 61% eosinophilia found after 6 OVA aerosols was much higher than those that are typically found in human asthmatics ([32]). Given the significant and prolonged parenchymal response to inhaled methacholine following 6 OVA aerosols and the level of eosinophilia present, it is likely that this model more closely parallels an allergic alveolitis ([33]) than the airway inflammation commonly seen in humans.

In recent studies there has been some focus on the association, or lack thereof, between indicators of systemic sensitisation, such as the levels of serum antibodies, airway inflammation and AHR ([34]). In a review of the role of IgE in the induction of eosinophilic airway inflammation and AHR, Hamelmann *et al*. ([35]) concluded that systemic methods of sensitisation resulted in high levels of IgE and eosinophilic airway inflammation in BALB/c mice. In these models, AHR was determined to be dependent on eosinophils but not IgE. However, the results of our study, which uses a similar protocol to those reviewed by Hamelmann *et al*. ([35]), show that the presence of eosinophils did not always coincide with an increase in responsiveness to MCh. Three OVA aerosols resulted in a significant eosinophilia after 24 hours but an increase in the response to the maximum dose of MCh was not evident until 48 hours post challenge. In contrast, a single OVA challenge resulted in hyperresponsiveness to MCh that had resolved by 48 hours while the levels of eosinophils remained significantly elevated. The levels of total serum IgE were equivalent across all challenge doses suggesting that, while the presence of IgE may be necessary to initiate the allergic response, its presence at a particular measurement time point does not necessarily relate to the presence of hyperresponsivenss.

## Conclusions

The findings of the present study demonstrate the significant impact of changing antigen challenge dose in a murine model of allergic bronchopulmonary inflammation. Given the variability of the inflammatory profile and characteristic responses observed in this study, it is clear that investigators must carefully characterise their allergen-driven murine models to ensure the model used contains the characteristic of interest. Future studies need to be directed at understanding the mechanisms that underlie airway and parenchymal hyperresponsiveness post antigen challenge.

## Authors' contributions

GRZ carried out the animal studies and drafted the manuscript. CvG carried out the IgE analysis and assisted in the interpretation of results and editing the manuscript. PAS assisted in the interpretation of results and editing the manuscript. PGH assisted in the conceptualisation of the study and interpretation of the results. PDS and DJT assisted in the conceptualisation of the study, interpretation of the results and editing the manuscript.
